# CircPRKCA Promotes NSCLC Progression via miR-200b-3p/FRMD6/SNAI2 Axis

**DOI:** 10.3390/ijms27093824

**Published:** 2026-04-25

**Authors:** He Zhong, Ning Wang, Hui Zhang, Min Chen, Xin Liao, Chao Huang

**Affiliations:** 1 Department of Urology, Union Hospital, Tongji Medical College, Huazhong University of Science and Technology, Wuhan 430022, China; 2 Department of General Practice, Tongji Hospital, Tongji Medical College, Huazhong University of Science and Technology, Wuhan 430030, China

**Keywords:** circPRKCA, non-small cell lung cancer, miR-200b-3p

## Abstract

Circular RNAs (circRNAs) have been reported to be closely associated with tumor progression in multiple malignancies. However, the specific mechanism by which circPRKCA influences tumor progression has not been fully elucidated. CircPRKCA is highly expressed in non-small cell lung cancer (NSCLC) tissues and cells. Knockdown of circPRKCA inhibits the malignant behaviors of NSCLC cells. RNA sequencing results revealed that *FRMD6* and *SNAI2* mRNAs are positively correlated with circPRKCA. Subsequently, we proved that circPRKCA acts as a molecular sponge for miR-200b-3p. Additionally, miR-200b-3p binds to the 3′ untranslated regions (3′UTRs) of *FRMD6* and *SNAI2* mRNAs to promote their degradation. Overexpression of circPRKCA thereby suppresses this degradation process and coun-teracts the tumor-suppressive effects induced by miR-200b-3p. CircPRKCA functions as the sponge of miR-200b-3p, suppressing the *SNAI2*/*FRMD6* mRNA degradation driven by miR-200b-3p and accelerating NSCLC progression.

## 1. Introduction

Circular RNAs (circRNAs) represent a unique class of covalently closed endogenous transcripts that function predominantly as molecular sponges for microRNAs (miRNAs), thereby modulating the expression of downstream target genes [1,2,3]. Among these, hsa_circ_0007580 (termed circPRKCA), derived from the PRKCA gene, has emerged as a critical regulator in non-small cell lung cancer (NSCLC) [4]. While a recent study has underscored the clinical significance of circPRKCA in promoting NSCLC progression, the intricate molecular networks and alternative signaling axes through which it exerts its oncogenic effects remain to be fully mapped. Understanding these unresolved mechanisms is crucial for identifying precise therapeutic targets in circPRKCA-driven malignancies.

The Hippo signaling pathway, a highly conserved evolutionary cascade, serves as a fundamental governor of cell proliferation and apoptosis. Dysregulation of this pathway, characterized by the aberrant activation of its key downstream effectors, YAP and TAZ, is frequently observed in NSCLC and correlates with poor prognosis [5,6,7,8]. Emerging evidence suggests that circRNAs can act as upstream modulators of the Hippo kinase core, either by sequestering pathway-specific miRNAs or through direct protein interactions [9,10,11]. However, whether circPRKCA engages the Hippo signaling axis to drive the malignant phenotype of NSCLC cells remains an intriguing and unexplored question.

In this study, through high-throughput transcriptome sequencing of NSCLC patient tissues, we identified circPRKCA as a significantly upregulated circular RNA. Clinical correlation analysis and functional assays revealed that circPRKCA acts as a molecular sponge to sequester miR-200b-3p, thereby relieving the inhibitory effect on SNAI2 and FRMD6 expression. This molecular decoy mechanism leads to the constitutive activation of the Hippo signaling pathway, which subsequently drives the malignant progression of NSCLC. Collectively, our findings delineate a novel circPRKCA/miR-200b-3p/Hippo regulatory axis and highlight circPRKCA as a potential diagnostic biomarker and therapeutic vulnerability in NSCLC.

## 2. Results

### 2.1. Identification and Characterization of circPRKCA in Lung Cancer

**Figure 1:**
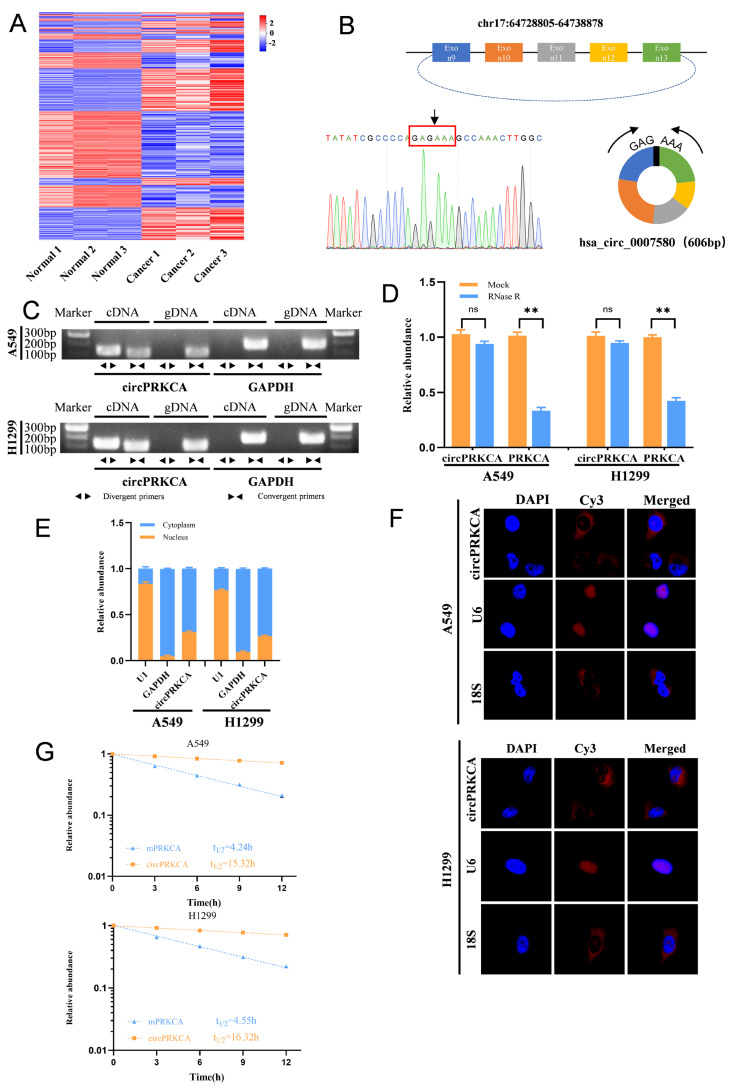
(**A**) Heatmap depicted the differentially expressed circRNAs in paired tumors and normal tissues contained three independent replicates, respectively. (**B**) Scheme illustrating the production of circPRKCA. Sequencing analysis of head-to-tail splicing junction in circPRKCA. (**C**) The existence of circPRKCA was validated in A549 and H1299 lung cancer cell lines by qRT-qPCR. Divergent primers amplified circPRKCA in cDNA but not genomic DNA (gDNA). GAPDH was used as negative control. (**D**) The relative RNA levels were analyzed by RT-qPCR in A549 and H1299 cells treated with or without RNase R. (**E**) Identification of circPRKCA cytoplasmic and nuclear distribution by RT-qPCR analysis in A549 and H1299 cells. GAPDH and U1 were applied as positive controls in the cytoplasm and nucleus, respectively (*n* = 3). (**F**) Identification of circPRKCA cytoplasmic and nuclear distribution by FISH in A549 and H1299 cells. 18S and U6 were applied as positive controls in the cytoplasm and nucleus, respectively; circPRKCA, 18S, and U6 probes were labeled with Cy3; nuclei were stained with DAPI. (**G**) After treatment with actinomycin D (5 μg/mL), the relative RNA levels of circPRKCA and mPRKCA in A549 and H1299 cells were analyzed by qRT-PCR (*n* = 3). Data: mean ± SD, *n* = 3. ns, not significant, ** *p* < 0.01 (Student’s *t*-test).

To identify key circRNAs involved in NSCLC, we performed high-throughput transcriptome sequencing on human lung cancer tissues and their paired adjacent normal tissues. Differentially expressed circRNAs were visualized using a heatmap (Figure 1A), among which hsa_circ_0007580 (termed circPRKCA) emerged as the most significantly upregulated candidate. Based on the human reference genome (GRCh37/hg19), circPRKCA is derived from exons 9–13 of the PRKCA gene located on chromosome 17. The circular structure of circPRKCA, with a predicted length of 606 bp, was validated by Sanger sequencing, which confirmed the presence of the unique back-splice junction (Figure 1B). Subsequent RT-qPCR assays using convergent and divergent primers demonstrated that circPRKCA could be amplified exclusively from complementary DNA (cDNA) but not from genomic DNA (gDNA) (Figure 1C). Furthermore, circPRKCA exhibited marked resistance to RNase R exonuclease digestion, further substantiating its covalently closed circular configuration (Figure 1D). Moreover, nucleocytoplasmic fractionation and FISH assays revealed that circPRKCA is predominantly localized within the cytoplasm (Figure 1E,F), suggesting its potential role in post-transcriptional gene regulation. Finally, circPRKCA transcripts were more stable compared with PRKCA mRNA after treating with actinomycin D (Figure 1G).

### 2.2. CircPRKCA Promotes Malignant Phenotypes in NSCLC Cells

**Figure 2:**
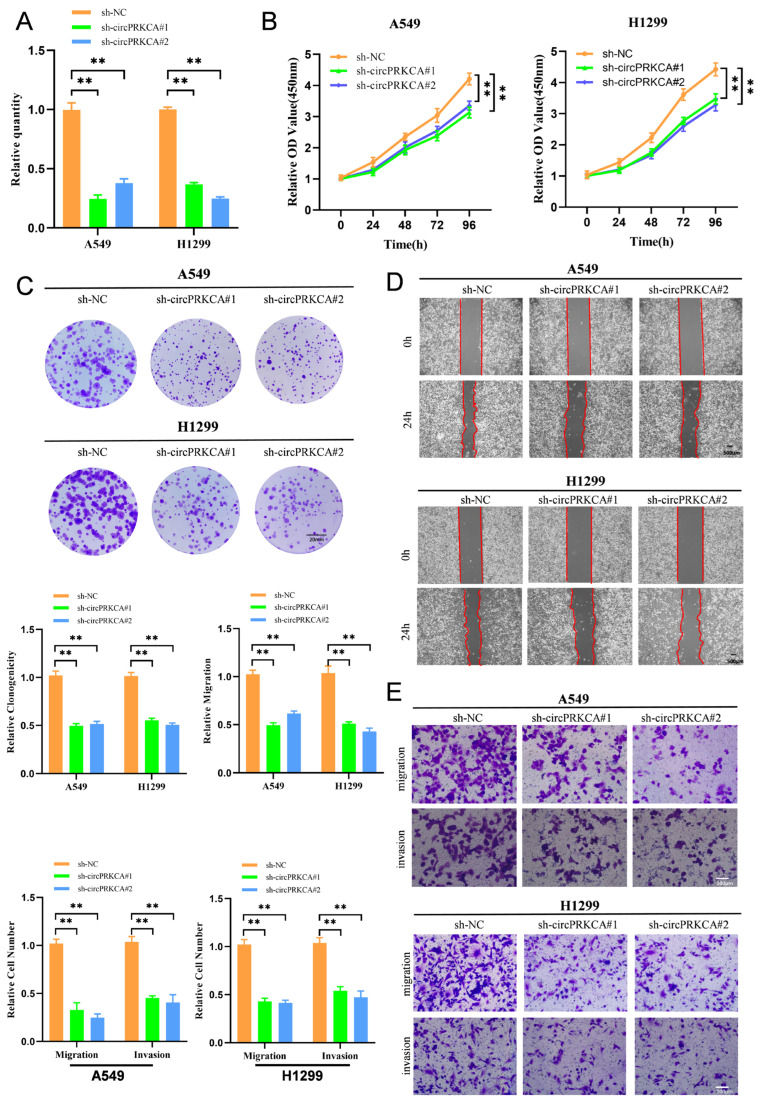
(**A**) The expression of circPRKCA was detected by RT-qPCR in A549 and H1299 cells stably transfected with sh-NC, sh-circPRKCA #1, or sh-circPRKCA #2. (**B**) Cell Counting Kit-8 assay revealed the cell viability of A549 and H1299 cells stably transfected with sh-NC, sh-circPRKCA #1, or sh-circPRKCA #2. (**C**) Colony formation assay in A549 and H1299 cells stably transfected with sh-NC, sh-circPRKCA #1, or sh-circPRKCA #2. (**D**) The wound healing capacity of A549 and H1299 cells transfected with sh-NC, sh-circPRKCA #1, or sh-circPRKCA #2. (**E**) Representative images and quantification of Transwell assay indicating the proliferation and invasion of A549 and H1299 cells. Scale bars: 20 mm (**C**), 500 μm (**D**), 100 μm (**E**). Data are presented as the means ± SD from three independent experiments. **, *p* < 0.01 (ANOVA).

**Figure 3:**
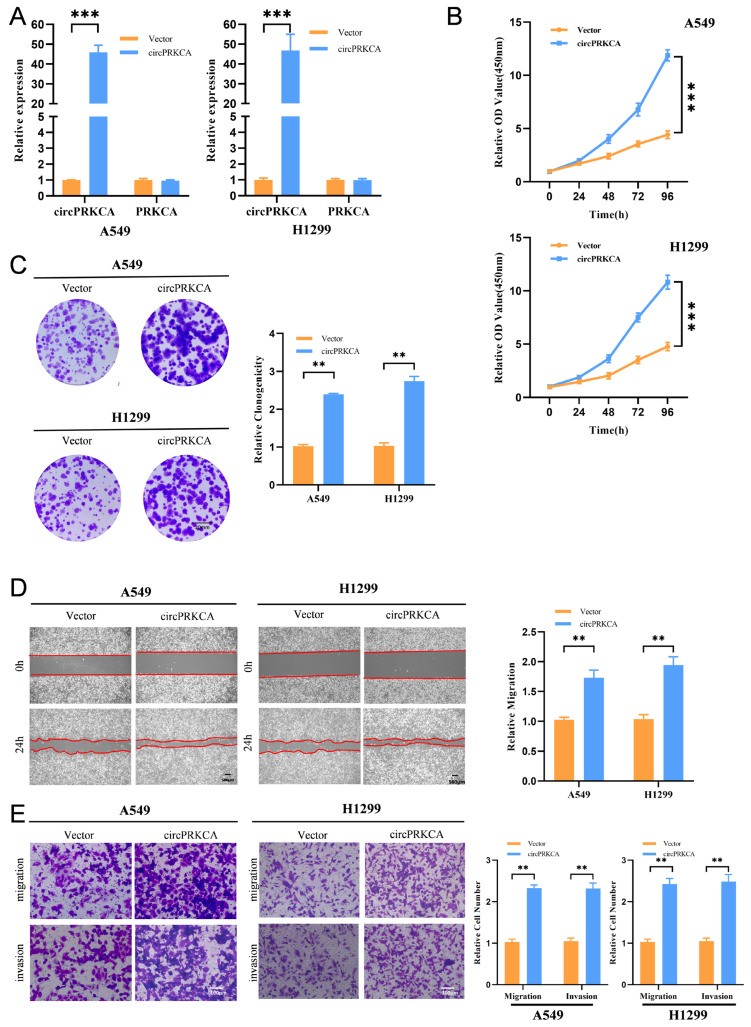
(**A**) Overexpression efficiency of circ-PRKCA in A549 and H1299 cells was verified by RT-qPCR. (**B**) Cell Counting Kit-8 assay revealed the cell viability of A549 and H1299 cells stably overexpressing circPRKCA. (**C**–**E**) Representative images and quantification of the colony formation, migration, and invasion of A549 and H1299 cells by Colony formation, Wound healing and Transwell assays. Scale bars: 20 mm (**C**), 500 μm (**D**), 100 μm (**E**). Data are presented as the means ± SD from three independent experiments. **, *p* < 0.01, ***, *p* < 0.001 (Student *t* test).

To evaluate the biological role of circPRKCA in NSCLC, A549 and H1299 cells were transfected with two independent short hairpin RNA (shRNA) constructs (Figure 2A). Functional assays demonstrated that circPRKCA silencing markedly impaired NSCLC cell growth, as reflected by reduced proliferative and clonogenic capacities (Figure 2B,C). In parallel, depletion of circPRKCA significantly compromised the migratory and invasive abilities of lung cancer cells in vitro (Figure 2D,E). Conversely, CircPRKCA upregulation robustly enhanced cellular proliferation and clonogenic potential (Figure 3A–C), accompanied by a pronounced increase in cell migration and invasion (Figure 3D,E).

### 2.3. CircPRKCA Upregulates FRMD6 and SNAI2 Expression in NSCLC Cells

**Figure 4:**
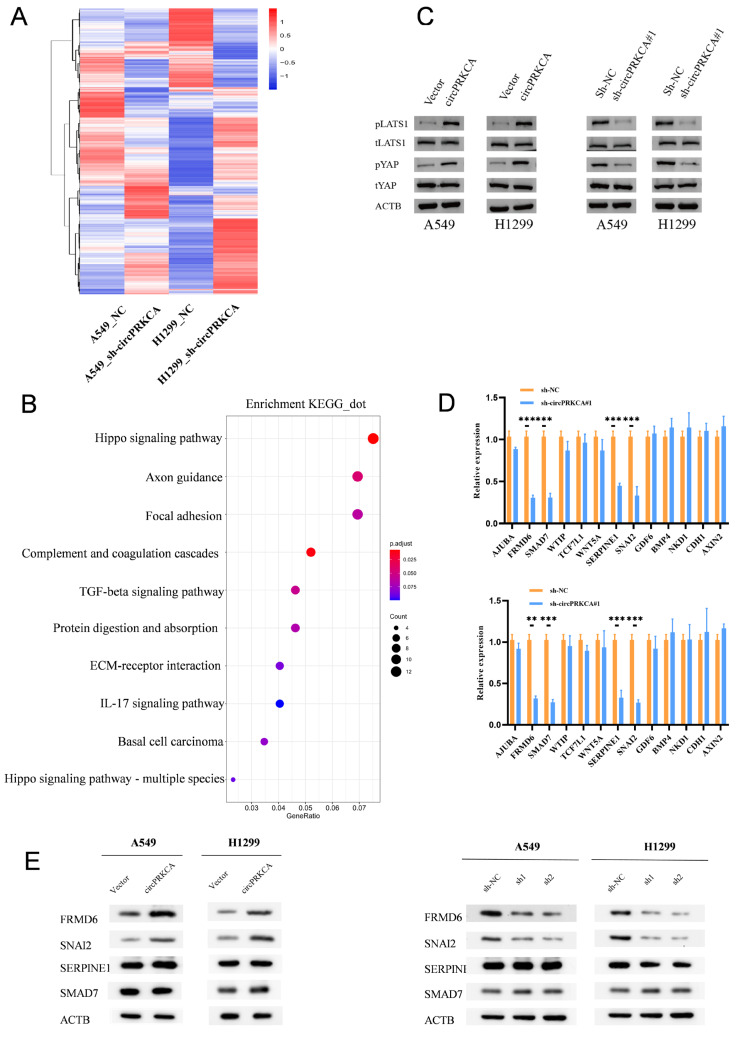
(**A**) Heatmap depicted the differentially expressed mRNAs upon circPRKCA knockdown in A549 and H1299 cells. (**B**) KEGG enrichment analysis revealed the enriched pathways upon circPRKCA knockdown in A549 and H1299 cells. (**C**) Western blotting with the indicated antibodies in A549 and H1299 cells upon circPRKCA knockdown or overexpression. ACTB was used as an internal control. (**D**) The expression of different genes enriched in Hippo signaling pathway was detected by RT-PCR in A549 and H1299 cells. (**E**) Western blotting with the indicated antibodies in A549 and H1299 cells upon circPRKCA knockdown or overexpression. ACTB was used as an internal control. Data are presented as the means ± SD from three independent experiments. **, *p* < 0.01, ***, *p* < 0.001 (Student *t* test).

To further explore the molecular pathways regulated by circPRKCA, we performed transcriptome clustering analysis on A549 and H1299 cells following circPRKCA knockdown (Figure 4A). KEGG enrichment analysis of the overlapping downregulated genes identified the Hippo signaling pathway as the most significantly enriched cascade (Figure 4B). Western blot analysis further validated the activation of the Hippo pathway (Figure 4C). Subsequent RT-qPCR validation of key Hippo-related candidates showed that SERPINE1, SNAI2, SMAD7, and FRMD6 were transcriptionally downregulated upon circPRKCA depletion (Figure 4D). However, Western blot analysis revealed that only FRMD6 and SNAI2 exhibited significant changes at the protein level consistent with the circRNA expression (Figure 4E). Therefore, we focused on FRMD6 and SNAI2 as the primary functional effectors of circPRKCA for further investigation.

### 2.4. CircPRKCA Promotes NSCLC Progression Through the FRMD6/SNAI2 Expression

**Figure 5:**
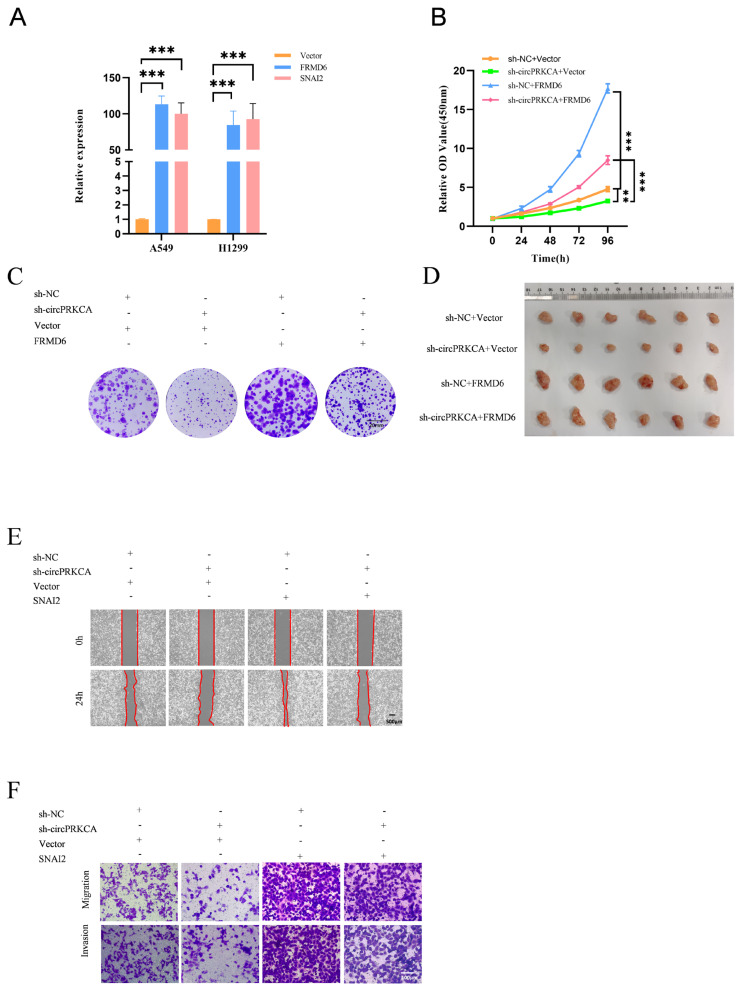
(**A**) The expressions of FRMD6 and SNAI2 were detected by RT-qPCR in A549 and H1299 cells stably overexpressing FRMD6 or SNAI2. (**B**–**F**) Rescue experiments confirmed the changes in proliferation, colony formation, migration, in vivo growth and invasion of A549 and H1299 cells. (**D**) in vivo growth representative of xenograft tumors formed by subcutaneous injection of the indicated A549 cells into the BALB/c-nu nude mice. Scale bars: 20 mm (**C**), 500 μm (**E**), 100 μm (**F**). Data are presented as the means ± SD from three independent experiments. **, *p* < 0.01; ***, *p* < 0.001 (ANOVA).

To determine whether FRMD6 and SNAI2 mediate the tumor-suppressive effects induced by circPRKCA knockdown, we ectopically overexpressed FRMD6 or SNAI2 in circPRKCA-knockdown A549 and H1299 cells (Figure 5A). The restoration of FRMD6 expression significantly rescued the impaired proliferative and clonogenic capacities caused by circPRKCA knockdown (Figure 5B,C). Subsequently, to validate these findings in vivo, we established subcutaneous xenograft models, which demonstrated that FRMD6 and SNAI2 restoration rescued the diminished tumor-forming potential of circPRKCA-knockdown cells (Figure 5D and Figure S1). Similarly, SNAI2 overexpression markedly reversed the suppression of cell migration and invasion induced by circPRKCA knockdown (Figure 5E,F). Collectively, these findings establish that circPRKCA drives NSCLC progression through the coordinated regulation of FRMD6 and SNAI2.

### 2.5. miR-200b-3p Functioned as the Sponge of circPRKCA in NSCLC Cells

**Figure 6:**
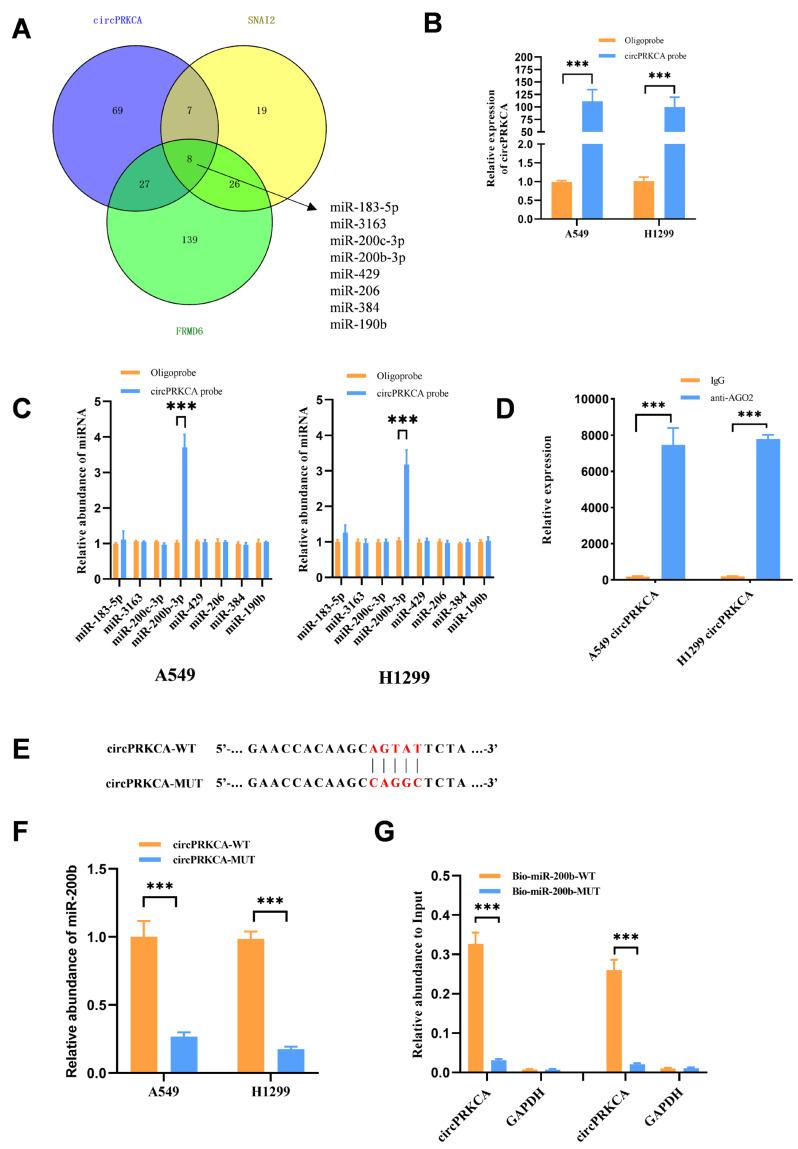
(**A**) The miRNAs binding to circPRKCA and FRMD/SNAI2 mRNAs were predicted by the starBase v2.0 database. (**B**,**C**) Using the circPRKCA probe to pull down RNAs, miR-200b-3p was identified as binding to circPRKCA. (**D**) AGO2 RNA immunoprecipitation (RIP) assay was performed to exclude false-positive binding between circPRKCA and miR-200b-3p. (**E**,**F**) A mutant of circPRKCA was designed. (**G**) A biotinylated mutant of miR-200b-3p was used to identify the binding with circPRKCA. GAPDH was used as an internal control. Data are presented as the means ± SD from three independent experiments. ***, *p* < 0.001 (Student’s *t* test).

Given that circPRKCA predominantly localized in the cytoplasm, we explored whether circPRKCA influenced malignant behaviors of tumors by sponging miRNAs. Using the online tool [12], eight miRNAs potentially associated with circPRKCA and SNAI2 and FRMD6 mRNAs were predicted (Figure 6A). To validate whether circPRKCA could bind to these candidate miRNAs, we performed an RNA pull-down assay with the circPRKCA probe (Figure 6B,C). As shown in Figure 6C, only miR-200b-3p was significantly enriched in the circPRKCA probe group. AGO2 RIP assay was conducted to verify the specific interaction between circPRKCA and miR-200b-3p and rule out false-positive results (Figure 6D). To further verify the specificity of this interaction, a mutant circPRKCA construct was designed (Figure 6E). As expected, wild-type (WT) circPRKCA captured a substantially higher amount of endogenous miR-200b-3p compared to the mutant version (Figure 6F). Furthermore, a reciprocal pull-down using biotinylated miR-200b-3p mimics demonstrated that the mutant miRNA exhibited a markedly impaired capacity to enrich circPRKCA compared to the WT mimic (Figure 6G). These results collectively confirm a direct interaction between miR-200b-3p and circPRKCA.

### 2.6. miR-200b-3p Interacts with the 3′ Untranslated Regions (3′UTRs) of FRMD6 and SNAI2 mRNA

**Figure 7:**
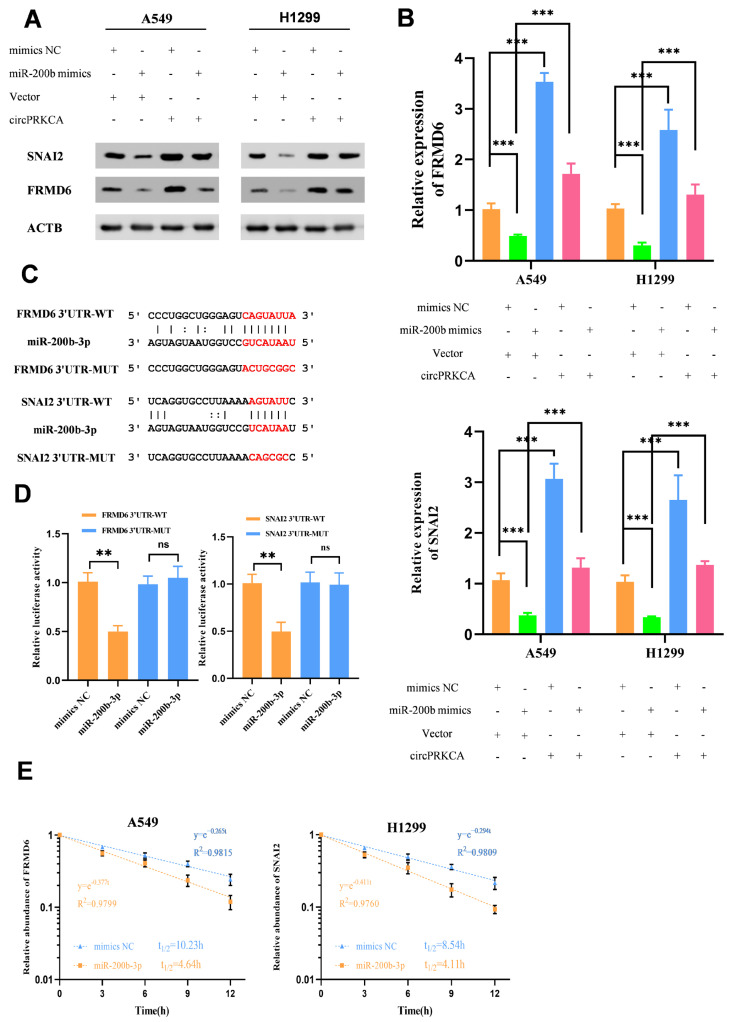
(**A**,**B**) Rescue experiments confirmed the protein level changes in SNAI2 and FRMD6 via Western blot. (**C**) The binding sites between miR-200b-3p and SNAI2/FRMD mRNAs 3′UTR were predicted by the starBase v2.0 database. (**D**) SNAI2/FRMD mRNAs 3′UTR mutants showed decreased circPRKCA enrichment ability. (**E**) The relative remaining levels of miR-200b-3p were analyzed by RT-qPCR after treatment with actinomycin D (5 μg/mL) at the indicated time points in A549 or H1299 cells transfected with mimics NC or miR-200b-3p. Data are presented as the means ± SD from three independent experiments. ns—not significant, **, *p* < 0.01; ***, *p* < 0.001 (Student’s *t* test, ANOVA).

In order to further corroborate the ceRNA network, we employed synthetic miR-200b-3p mimics, which successfully abrogated the upregulation of FRMD6 and SNAI2 protein levels induced by circPRKCA overexpression (Figure 7A,B). To explore the regulatory mechanism of miR-200b-3p in NSCLC progression, we predicted the alignments between miR-200b-3p and FRMD6/SNAI2 mRNAs (Figure 7C). Dual-luciferase reporter assays indicated these alignments (Figure 7D). Upon actinomycin D treatment, FRMD6 and SNAI2 transcripts were less stable with exogenous miR-200b-3p mimics (Figure 7E).

### 2.7. The Oncogenic Effects of circPRKCA Are Suppressed by miR-200b-3p

**Figure 8:**
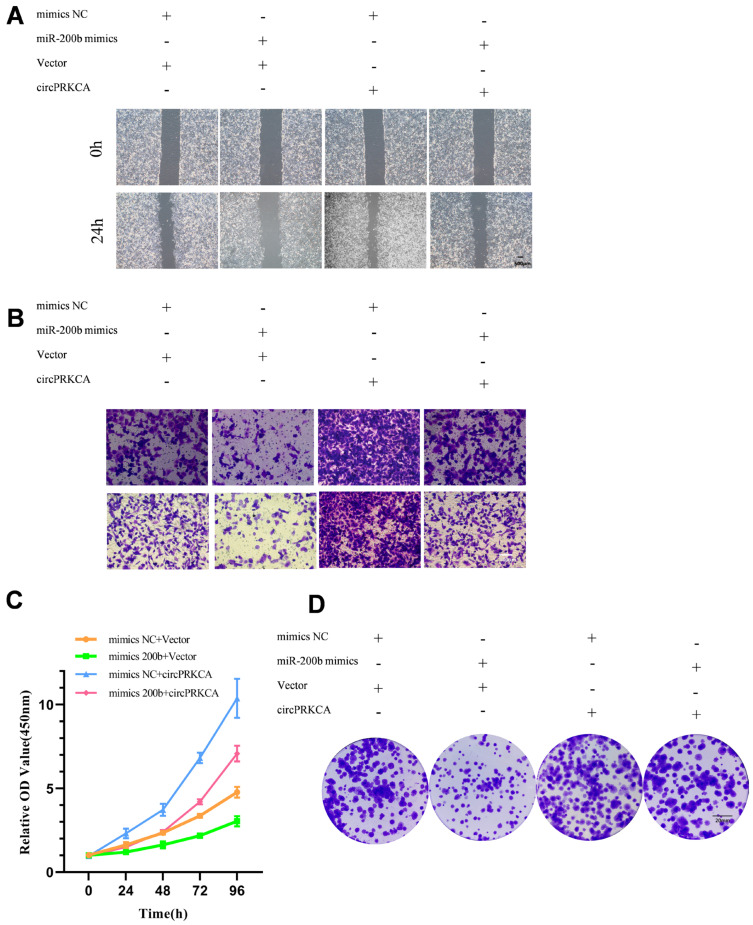
(**A**–**D**) Rescue experiments confirmed the changes in the migration, invasion, proliferation and colony formation by Wound healing, Colony formation, CCK-8 and Transwell assays in A549 and H1299 cells. Scale bars: 20 mm (**A**), 500 μm (**B**), 100 μm (**D**).

To further investigate whether the oncogenic functions of circPRKCA are regulated by miR-200b-3p, we performed a series of rescue experiments. The increased migratory capacity driven by circPRKCA was substantially attenuated by miR-200b-3p (Figure 8A), accompanied by a pronounced reduction in invasive ability (Figure 8B). Moreover, miR-200b-3p significantly impaired the circPRKCA-induced enhancement of cell proliferation and clonogenic capacity (Figure 8C,D).

## 3. Discussion

Circular RNAs (circRNAs), characterized by their covalently closed-loop structure and remarkable stability, have emerged as pivotal regulatory elements in the landscape of tumorigenesis and malignant progression [13,14,15,16]. Therefore, elucidating the intricate functional networks of circRNAs is paramount for deciphering the molecular etiology of lung cancer and identifying novel therapeutic vulnerabilities. In the present study, through high-throughput transcriptome sequencing of lung cancer tissues, we identified circPRKCA as significantly upregulated in lung cancer cells. In vitro and in vivo evidence demonstrated that circPRKCA promotes cell proliferation and invasion, supporting its role as a potent oncogenic driver in lung cancer. Mechanistically, circPRKCA serves as a molecular sponge for miR-200b-3p, effectively abrogating miR-200b-3p-mediated degradation of FRMD6 and SNAI2 mRNAs, thereby contributing to tumor progression. In conclusion, our study suggests that circPRKCA could serve as a potential diagnostic biomarker and a promising therapeutic target for intervention in lung cancer patients.

CircRNAs are ubiquitously expressed genes characterized by their precise regulatory patterns, and they are prime candidates for deciphering the complexities of gene expression control in human tissues. Accumulating evidence has implicated circRNAs in a wide range of human diseases, including cancer and [3,17,18]. In lung cancer, several circRNAs have been reported to modulate tumor progression through functioning as miRNA sponges or a protein-binding partner [19,20]. In this study, we identified that circPRKCA is significantly upregulated in NSCLC cells and primarily localizes within the cytoplasm. Gain- and loss-of-function assays demonstrated that circPRKCA markedly enhances the malignant phenotype of lung cancer cells. Although a previous study reported that curcumin suppresses the NSCLC growth by downregulating circPRKCA [4], our work systematically elucidates the downstream regulatory network of circPRKCA using high-throughput sequencing approaches. Notably, our findings reveal that circPRKCA promotes NSCLC progression via the Hippo signaling pathway, thereby uncovering a novel mechanistic dimension. Although we confirmed its cytoplasmic function as a miRNA sponge, the potential of circPRKCA to encode functional peptides remains an area for future investigation.

FRMD6 is typically recognized as an upstream activator of the mammalian Hippo pathway, acting as a scaffold protein that recruits and activates the MST1/2-LATS1/2 kinase complex [21]. This cascade promotes the phosphorylation and cytoplasmic sequestration of YAP/TAZ, thereby preventing their nuclear translocation and transcriptional activity. While high FRMD6 expression correlates with a favorable prognosis and tumor suppression in prostate and colorectal cancers, its role appears to be context-dependent. In certain cancers such as lung cancer, FRMD6 has been shown to promote tumor cell proliferation, migration, and growth by interacting with and activating the mTOR/S6K signaling axis. Our findings further substantiate the pro-tumor role of FRMD6 in lung cancer. The complex duality of FRMD6 may be dependent on cellular context and the tumor microenvironment. Moreover, the potential crosstalk between Hippo and mTOR signaling pathways further highlights the central role of circPRKCA in the malignant reprogramming of lung cancer cells. Unlike FRMD6, SNAI2 is not a core component of the Hippo pathway but can form a transcriptional complex with YAP/TAZ to drive downstream target expression [22]. Although the precise mechanistic interplay among circPRKCA, FRMD6, and SNAI2 requires further investigation, we identified circPRKCA as a molecular sponge for miR-200b-3p, thereby relieving miR-200b-3p-mediated degradation of both FRMD6 and SNAI2 mRNAs. Clinically, circPRKCA and its downstream axis molecules hold potential as promising diagnostic biomarkers or therapeutic targets for NSCLC. Targeting this regulatory axis may facilitate the development of more precise and effective strategies for lung cancer treatment, thereby advancing the field of circRNA-directed precision medicine in NSCLC.

## 4. Materials and Methods

### 4.1. Patients and Tissue Specimen Collection

Tissue samples were collected from the Department of Thoracic Surgery, Tongji Hospital, Tongji Medical College (Wuhan, China) between November 2023 and April 2025. With the informed consent of the patients, paired specimens of non-small cell lung cancer (NSCLC) and adjacent non-cancerous lung tissue were obtained from our medical institution, with 6 samples in total. The adjacent non-cancerous lung tissue was selected from the area 3 cm away from the tumor margin. According to the revised International Cancer Staging System, all patients were diagnosed with NSCLC based on their histological and pathological findings. None of these patients had received chemotherapy or radiotherapy prior to sample collection. All samples were rapidly frozen and stored in a −80 °C refrigerator for preservation.

### 4.2. Cell Culture

A549 and H1299 cells were purchased from American Type Culture Collection (ATCC, Virginia, MA, USA). Cells were maintained in Dulbecco’s modified Eagle’s medium (DMEM) supplemented with 10% FBS (Gibco, Australia origin, Gibco, Grand Island, NY, USA), 1% penicillin/streptomycin.

### 4.3. RNA Preparation, RNase R, and RT–qPCR

Total RNA was isolated from cells or tissues using the miRNeasy Mini Kit (Qiagen, Hilden, NRW, Germany), and nuclear and cytoplasmic RNA were extracted with the Nuclear and Cytoplasmic RNA Purification Kit (Fisher Scientific, Waltham, MA, USA, AM1921). For RNase R treatment, 1 μg total RNA was incubated at 37 °C for 15 min with or without 3 U RNase R (Epicentre Technologies, Madison, WI, USA). To validate circRNA backspliced junctions, total RNA was treated with the RiboZero rRNA Removal Kit (Epicentre, Madison, WI, USA) to deplete rRNA (per the manufacturer’s instructions), followed by cDNA synthesis from rRNA-depleted and RNase R-digested RNA using random primers (Takara, Dalian, China). For mRNA and circRNA quantification, cDNA was synthesized from 500 ng RNA with the PrimeScript RT Master Mix (Takara). Real-time PCR was performed with SYBR Premix Ex Taq II (Takara), using divergent primers (annealing at circRNA distal ends) to determine circRNA abundance (primers listed in Supplementary Table S1). Amplification was conducted on the StepOnePlus Real-Time PCR System (Applied Biosystems, Foster City, CA, USA), with Ct thresholds determined by software.

### 4.4. Actinomycin D Treatment and RNA Stability Assay

Cells were seeded in 6-well plates to 70% confluency and treated with 5 μg/mL Actinomycin D (Sigma-Aldrich, St. Louis, MO, USA), a transcription inhibitor; untreated cells served as controls. At 0, 2, 4, 6 and 8 h post-treatment, total RNA was extracted with TRIzol reagent (Invitrogen, Waltham, MA, USA), and its purity (A260/A280 = 1.8–2.1) was verified by NanoDrop 2000 (Thermo Fisher Scientific, New York, NY, USA). First-strand cDNA was synthesized from 1 μg total RNA, and qRT-PCR was performed with gene-specific primers and SYBR Green Master Mix. Target RNA expression was normalized to 18S rRNA. RNA half-life (t_1_/_2_) was calculated by fitting expression data to a one-phase exponential decay model using GraphPad Prism 9. All experiments were performed in triplicate with three independent biological repeats.

### 4.5. RNA Immunoprecipitation (RIP) Assay

RNA immunoprecipitation (RIP) assays were executed utilizing the Magna RIP™ RNA-Binding Protein Immunoprecipitation Kit (Millipore, Burlington, MA, USA) in strict accordance with the manufacturer’s protocols. Briefly, NSCLC cells were lysed using RIP lysis buffer supplemented with protease and RNase inhibitors. The resulting lysates were incubated with magnetic beads conjugated to anti-AGO2 antibody or normal IgG (negative control) at 4 °C overnight under gentle rotation. Following extensive washing, protein–RNA complexes were eluted, and RNA was extracted. The enrichment of circPRKCA and miR-200b-3p within the AGO2 immunoprecipitates, relative to the IgG control, was subsequently quantified via qRT-PCR.

### 4.6. RNA Pull-Down Assays

Biotin-labeled circPRKCA (sense) and control (antisense) probes were synthesized by TSINGKE (Wuhan, China). RNA pull-down assays were performed as previously described [9]. Briefly, 10^7^ cells were washed with ice-cold phosphate-buffered saline, lysed in 500 μL Co-IP buffer (20 mM Tris-HCL, pH 7.5, 150 mM NaCl, 1 mM EDTA, 0.5% NP-40, complete protease inhibitor cocktail and RNase inhibitors), and incubated with 3 μg biotinylated DNA oligo probes at room temperature for 2 h. Then 50 μL washed Streptavidin C1 magnetic beads (Invitrogen) were added to each binding reaction and incubated for another 1 h at room temperature. The beads were briefly washed five times with Co-IP buffer, and the bound proteins in the pull-down products were analyzed by mass spectrometry or Western blotting.

### 4.7. Western Blot

Whole-cell lysates were prepared with RIPA buffer containing protease inhibitors (Beyotime, Shanghai, China). After boiling, supernatants were subjected to SDS-PAGE and transferred to nitrocellulose membranes. Following blocking with 5% non-fat milk, membranes were sequentially incubated with primary and HRP-conjugated secondary antibodies, and bands were visualized using enhanced chemiluminescence (E412–01, Vazyme, Nanjing, China). The antibodies used were: primary antibodies against LATS1 (ab70562 Abcam, Cambridge, UK), phosphorylated LATS1 (Thr1079, ab111344 Abcam, Cambridge, UK), YAP1 (ab52771 Abcam, Cambridge, UK), and phosphorylated YAP1 (Ser127, ab76252 Abcam, Cambridge, UK), FRMD6 (ab171745, Abcam, Cambridge, UK), SNAI2 (ab27568, Abcam, Cambridge, UK), SMAD7 (ab216428, Abcam, Cambridge, UK), SERPINE1 (13801-1-AP, Proteintech, Rosemont, IL, USA), and β-Actin (ab6726, Abcam, Cambridge, UK); HRP-conjugated secondary antibodies included goat anti-mouse (ab6789, Abcam, Cambridge, UK) and goat anti-rabbit (ab6721, Abcam, Cambridge, UK).

### 4.8. Fluorescent In Situ Hybridization

Cells were fixed, dehydrated, and hybridized with specific FISH probes for the target RNA at 37 °C overnight. After stringency washing, samples were stained with DAPI for nuclear counterstaining. Fluorescent signals were captured under a laser scanning confocal microscope and analyzed using ImageJ(1.54p) software. All experiments were performed in triplicate.

### 4.9. Vector Construction and Cell Transfection

To construct circPRKCA, FRMD6 and SNAI2 overexpression plasmids, human circPRKCA, FRMD6, SNAI2 and p73 cDNAs were synthesized by TSINGKE (Wuhan, China) and cloned into pcDNA3.1(+) CircRNA Mini Vector (addgene #60648) and p3XFLAG-CMV-10 vector (Sigma-Aldrich), respectively. FRMD6 and SNAI2 truncations were amplified with specific primers and subcloned into the p3XFLAG-CMV-10 vector. Oligonucleotides encoding circPRKCA-specific short hairpin RNAs (shRNAs) were cloned into pLKO.1-puro (Sigma-Aldrich). Transfection was performed using Lipofectamine 2000 (Life Technologies, Carlsbad, CA, USA) following the manufacturer’s instructions. Stable cell lines were screened with neomycin or puromycin (Invitrogen). Empty vector and scramble shRNA (sh-Scb) were used as controls.

### 4.10. Colony-Formation Assay

The clonogenic capacity of cells was evaluated by a colony formation assay. Briefly, cells were trypsinized, counted, and seeded into 6-well plates at a low density (500–1000 cells per well, depending on the cell line) to allow distinct colony formation. Cells were then cultured in complete medium for 10–14 days, with the medium refreshed every 3–4 days. Subsequently, cells were washed twice with phosphate-buffered saline (PBS), fixed with 4% paraformaldehyde for 20 min, and stained with 0.1% crystal violet solution at room temperature for 30 min. After thorough washing with distilled water to remove excess dye, plates were air-dried. Colonies with more than 50 cells were manually counted under a microscope, and the experiment was performed in triplicate.

### 4.11. Wound-Healing Assay

Cell migration was assessed by a wound healing (scratch) assay. Cells were seeded into 12-well plates and cultured to 90–95% confluency. A sterile 200 μL pipette tip was used to create a straight scratch across the cell monolayer, and dislodged cells were removed by washing twice with PBS. Fresh serum-free medium was added to minimize the impact of cell proliferation. Wound area images were captured at 0 and 24 h using an inverted phase-contrast microscope (Olympus IX73, Olympus Corporation, Tokyo, Japan) with a digital camera. Migratory ability was quantified by measuring the wound width change at three predetermined positions per well using ImageJ software, and each experiment was conducted in triplicate.

### 4.12. Transwell Assay

Cell migration and invasion were determined using Transwell chambers (8-μm pore size, Corning, NY, USA). For the migration assay, 2–5 × 10^4^ cells in 200 μL serum-free medium were seeded into the upper chamber. For the invasion assay, the upper chamber was pre-coated with 50–100 μL Matrigel (BD Biosciences, San Jose, CA, USA) and allowed to solidify at 37 °C for 4 h before cell seeding. The lower chamber was filled with 500 μL complete medium containing 10% fetal bovine serum as a chemoattractant. After incubation at 37 °C for 24–48 h, non-migratory/non-invasive cells on the upper membrane surface were carefully removed with a cotton swab. Cells that migrated or invaded to the lower surface were fixed with 4% paraformaldehyde, stained with 0.1% crystal violet, and photographed under an inverted microscope. The number of cells in five random fields per well was counted for quantification, and all assays were performed in triplicate.

### 4.13. Nuclear and Cytoplasmic Extraction

Cytoplasmic and nuclear fractions were isolated according to the manufacturer’s instructions using reagents from the PARIS™ Kit (AM1556, Thermo Fisher Scientific, Waltham, MA, USA). Briefly, cells were lysed in Cell Fraction Buffer on ice for 10 min, centrifuged at 500× *g* for 3 min at 4 °C, and the supernatant was collected as the cytoplasmic fraction. The pellet was then washed with Cell Fraction Buffer, and nuclei were collected.

### 4.14. Cell Counting Kit-8 (CCK-8) Assay

Cell proliferation was detected using the CCK-8 kit (HYCEZMNIO, Wuhan, China) following the manufacturer’s instructions. The optical density (OD) at 450 nm was measured with an automatic microplate reader (Synergy4; BioTek, Winooski, VT, USA).

### 4.15. Subcutaneous Xenograft Tumor Model

BALB/c-nu nude mice (4–6 weeks old, female) were randomly divided into experimental and control groups (*n* = 6 per group). Logarithmic-phase lung cancer cells (2 × 10^6^ cells in 100 μL PBS) were subcutaneously injected into the right axilla of each mouse. Tumor volume was measured every 3 days with calipers and calculated using the formula: V = (length × width^2^)/2. On day 28, mice were sacrificed, and subcutaneous tumors were dissected, weighed, and photographed.

### 4.16. Statistical Analyses

Statistical analyses were performed using GraphPad Prism 9 software. Measurement data were expressed as mean ± standard deviation (SD). One-way analysis of variance (ANOVA) was used for comparing differences among multiple groups, and Student’s *t*-test was applied for pairwise comparisons of paired samples. A *p* value < 0.05 was considered statistically significant.

## 5. Conclusions

In conclusion, our study demonstrates that circPRKCA is upregulated in NSCLC and facilitates tumor proliferation, invasion and metastasis both in vitro and in vivo. Briefly, circPRKCA acts as a competing endogenous RNA (ceRNA) to sponge miR-200b-3p, thus relieving its inhibitory effect on the downstream target FRMD6 and further regulating SNAI2-mediated malignant progression. These findings establish the novel circPRKCA/miR-200b-3p/FRMD6/SNAI2 axis as a critical regulator in NSCLC development and provide new insights into the molecular mechanisms underlying lung cancer metastasis. Clinically, circPRKCA represents a promising prognostic biomarker and potential therapeutic target, highlighting that targeting this regulatory axis may offer novel and precise strategies for the treatment of non-small cell lung cancer.

## Data Availability

The datasets supporting the conclusions of this article are included within the article. The sequencing data have been uploaded to the SRA database under the accession number PRJNA1450910.

## Supplementary Materials

The following supporting information can be downloaded at: https://www.mdpi.com/article/10.3390/ijms27093824/s1.

## Abbreviations

The following abbreviations are used in this manuscript:

CCK8	Cell Counting Kit-8
RT-qPCR	Reverse transcription–quantitative real-time polymerase chain reaction
WB	Western blot
FISH	Fluorescent in situ hybridization
shRNA	Short hairpin RNA
NSCLC	Non-Small Cell Lung Cancer
circRNA	Circular RNA
miRNA	Micro RNA
