# Supplementary material for: CircPRKCA Promotes NSCLC Progression via miR-200b-3p/FRMD6/SNAI2 Axis
Source: Int J Mol Sci. 2026 Apr 25;27(9):3824. doi: 10.3390/ijms27093824 (PMC13163951; doi:10.3390/ijms27093824)
Supplement: Supplementary file 1 [file ijms-27-03824-s001.zip › ijms-4177274-supplementary.pdf]

Supplementary Table S1

circPRKCA Divergent primer	F:5′—GTGCAAGGAACACATGATGG—3′
	R:5′—ACTCGGTCAAGGTTGTTGGA—3′
circPRKCA Convergent primers	F:5′—AAGGCTTCCAGTGCCAAGTT—3′
	R:5′—CTCCTGGGGTCATCAGTGTC—3′

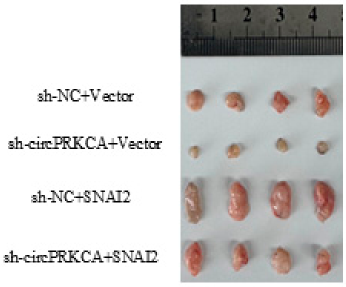

Figure S1 In vivo growth representative of xenograft tumors formed by subcutaneous injection of the indicated A549 cells into the BALB/c-nu nude mice (n = 4).
